# EXPLORING REASONS THAT U.S. MD-PHD STUDENTS ENTER AND LEAVE THEIR DUAL-DEGREE PROGRAMS

**DOI:** 10.28945/4622

**Published:** 2020

**Authors:** Devasmita Chakraverty, Donna B. Jeffe, Katherine P. Dabney, Robert H. Tai

**Affiliations:** Indian Institute of Management Ahmedabad, Ahmedabad, India; Washington University in St. Louis, St. Louis, MO, U.S.A.; Virginia Commonwealth University, Richmond, VA, U.S.A.; University of Virginia, Charlottesville, VA, U.S.A.

**Keywords:** MD-PhD program, Doctoral training challenges, Biomedical-research workforce, Attrition, Medical education

## Abstract

**Aim/Purpose:**

In response to widespread efforts to increase the size and diversity of the biomedical-research workforce in the U.S., a large-scale qualitative study was conducted to examine current and former students’ training experiences in MD (Doctor of Medicine), PhD (Doctor of Philosophy), and MD-PhD dual-degree programs. In this paper, we aimed to describe the experiences of a subset of study participants who had dropped out their MD-PhD dual-degree training program, the reasons they entered the MD-PhD program, as well as their reasons for discontinuing their training for the MD-PhD.

**Background:**

The U.S. has the longest history of MD-PhD dual-degree training programs and produces the largest number of MD-PhD graduates in the world. In the U.S., dual-degree MD-PhD programs are offered at many medical schools and historically have included three phases—preclinical, PhD-research, and clinical training, all during medical-school training. On average, it takes eight years of training to complete requirements for the MD-PhD dual-degree. MD-PhD students have unique training experiences, different from MD-only or PhD-only students. Not all MD-PhD students complete their training, at a cost to funding agencies, schools, and students themselves.

**Methodology:**

We purposefully sampled from 97 U.S. schools with doctoral programs, posting advertisements for recruitment of participants who were engaged in or had completed PhD, MD, and MD-PhD training. Between 2011-2013, semi-structured, one-on-one phone interviews were conducted with 217 participants. Using a phenomenological approach and inductive, thematic analysis, we examined students’ reasons for entering the MD-PhD dual-degree program, when they decided to leave, and their reasons for leaving MD-PhD training.

**Contribution:**

Study findings offer new insights into MD-PhD students’ reasons for leaving the program, beyond what is known about program attrition based on retrospective analysis of existing national data, as little is known about students’ actual reasons for attrition. By more deeply exploring students’ reasons for attrition, programs can find ways to improve MD-PhD students’ training experiences and boost their retention in these dual-degree programs to completion, which will, in turn, foster expansion of the biomedical-research-workforce capacity.

**Findings:**

Seven participants in the larger study reported during their interview that they left their MD-PhD programs before finishing, and these were the only participants who reported leaving their doctoral training. At the time of interview, two participants had completed the MD and were academic-medicine faculty, four were completing medical school, and one dropped out of medicine to complete a PhD in Education. Participants reported enrolling in MD-PhD programs to work in both clinical practice and research. Very positive college research experiences, mentorship, and personal reasons also played important roles in participants’ decisions to pursue the dual MD-PhD degree. However, once in the program, positive mentorship and other opportunities that they experienced during or after college, which initially drew candidates to the program was found lacking. Four themes emerged as reasons for leaving the MD-PhD program: 1) declining interest in research, 2) isolation and lack of social integration during the different training phases, 3) suboptimal PhD-advising experiences, and 4) unforeseen obstacles to completing PhD research requirements, such as loss of funding.

**Recommendations for Practitioners:**

Though limited by a small sample size, findings highlight the need for better integrated institutional and programmatic supports for MD-PhD students, especially during PhD training.

**Recommendations for Researchers:**

Researchers should continue to explore if other programmatic aspects of MD-PhD training (other than challenges experienced during PhD training, as discussed in this paper) are particularly problematic and pose challenges to the successful completion of the program.

**Impact on Society:**

The MD-PhD workforce comprises a small, but highly -trained cadre of physician-scientists with the expertise to conduct clinical and/or basic science research aimed at improving patient care and developing new diagnostic tools and therapies. Although MD-PhD graduates comprise a small proportion of all MD graduates in the U.S. and globally, about half of all MD-trained physician-scientists in the U.S. federally funded biomedical-research workforce are MD-PhD-trained physicians. Training is extensive and rigorous. Improving experiences during the PhD-training phase could help reduce MD-PhD program attrition, as attrition results in substantial financial cost to federal and private funding agencies and to medical schools that fund MD-PhD programs in the U.S. and other countries.

**Future Research:**

Future research could examine, in greater depth, how communications among students, faculty and administrators in various settings, such as classrooms, research labs, and clinics, might help MD-PhD students become more fully integrated into each new program phase and continue in the program to completion. Future research could also examine experiences of MD-PhD students from groups underrepresented in medicine and the biomedical-research workforce (e.g., first-generation college graduates, women, and racial/ethnic minorities), which might serve to inform interventions to increase the numbers of applicants to MD-PhD programs and help reverse the steady decline in the physician-scientist workforce over the past several decades.

## Introduction

Traditional doctoral training for the PhD involves time for trainees to learn to combine their knowledge of course content and research skills to produce original research, culminating with a doctoral dissertation (Lovitts, 2005). Typically, the average time of PhD-degree completion varies from 4-6 years (Bourke et al., 2004). The MD-PhD (Doctor of Medicine and Doctor of Philosophy) physician-scientist workforce comprises a relatively small cadre of well-trained physician-scientists with the research skills to address clinical and/or basic science research questions aimed at improving patient care (Goldstein & Kohrt, 2012; Varki & Rosenberg, 2002). In the U.S., MD-PhD training during medical school is extensive and lengthy, typically lasting for eight or more years (Brass et al., 2010; Jeffe et al., 2014a), and MD-PhD program attrition is a cause of concern. To our knowledge, only one study has been conducted to examine factors associated with MD-PhD program attrition (Jeffe et al., 2014a), and no studies have purposely examined MD-PhD students’ own reasons for leaving their MD-PhD program.

To fill a gap in the literature, we examined attrition from MD-PhD training programs in the U.S., where such training programs were first developed in the 1950s to increase the number of physician-scientists in the biomedical-research workforce (Harding et al., 2017) and where integrated dual-degree MD-PhD programs are the most prevalent. For the award period from July 1, 2019 through June 30, 2020, 50 of 154 U.S. Liaison Committee on Medical Education (LCME)-accredited medical schools had dual-degree MD-PhD programs that were funded by the U.S. National Institutes of Health National Institute of General Medical Sciences (NIH NIGMS) Medical Scientist Training Program (MSTP) (National Institute of General Medical Sciences, 2020). Many, if not all, MSTP-funded MD-PhD programs as well as non-MSTP-funded MD-PhD programs in U.S. medical schools receive training support from non-federal governmental and private funding organizations, other NIH institutes, and institutional funds to support MD-PhD training (AAMC, 2009; Jeffe et al., 2014a; Jeffe & Andriole, 2011). MD-PhD programs in other countries are small in number relative to the number of MD-PhD programs in the U.S. (Jones et al., 2016; Kuehnle et al., 2009; Twa et al., on behalf of the Canadian MD/PhD Program Investigation Group, 2017), and many of the nationally supported MD-PhD programs in other countries, such as Switzerland (Kuehnle et al, 2009) and Germany (Bossé et al., 2011), allow for PhD training to begin after receipt of the MD. A 2016-2017 survey of the European MD/PhD Association programs in multiple countries examined MD-PhD program characteristics in association with MD-PhD students’ and graduates’ opinions about the program, their career choices and outcomes (dos Santos Rocha et al., 2020); but we found no studies published that examined MD-PhD students’ self-reported reasons for leaving the MD-PhD program prior to completion.

This exploratory study therefore sought to answer the following research questions: “For MD-PhD students who discontinued their training, what motivated them to pursue MD-PhD training? Additionally, at what point during training and for what reasons did they discontinue their training?”

## Literature Review

### MD-PhD programs typically involve three phases:

two years of pre-clinical training in medical school, at least four years of PhD research training in graduate school, and two more years of clinical training after returning to medical school (Brass et al., 2010; Jeffe et al., 2014a). Acceptance to MD-PhD dual-degree programs is very competitive, and MD-PhD graduates have a greater planned career involvement in research at the time of medical-school graduation compared with all other MD graduates (Andriole et al., 2008), especially in disease-oriented and clinical research (Ahn et al., 2007; Andriole et al., 2008).

Not all students who matriculate into MD-PhD programs complete the program (Jeffe et al., 2014a; National Institutes of Health National Institute of General Medical Sciences [NIH-NIGMS], 1998). In an earlier survey study, more than one-fourth of enrolled MD-PhD students seriously considered leaving the program (Ahn et al., 2007). In a survey of 24 MD-PhD programs (Brass et al., 2010), attrition rates were reported to range from 3-34%. In a national cohort study of MD-PhD program enrollees at time of matriculation, the attrition rate was observed to be 27% (Jeffe et al., 2014a). By comparison, the attrition rate among MD-only students in the U.S. is about 3% (Association of American Medical Colleges [AAMC], 2012; Garrison et al., 2007). PhD enrollment and completion rates vary across universities, fields, countries, and demographic factors such as sex (Dabney et al., Tai, 2016); national-level data on PhD-program attrition is not well-documented. An Australian study collected data from approximately 1,200 students enrolled at one university to find a completion rate of 70% (Bourke et al., 2004). In another study, attrition data were collected in 2013-2014 in a survey of more than 1,500 psychology programs in the U.S. and found doctoral attrition rates between 5-13% (Michalski et al., 2016). Dropout rates during PhD training have been reported to be between 40% and 60% (Geiger, 1997; Tinto, 1987). The odds of PhD student dropout in STEM is most in the first year and greater for women (Lott et al., 2009). One study about underrepresented racial/ethnic minority (URM, including Black/African American, Hispanic/Latino, and American Indian/Native American) students in STEM that collated PhD completion rates for ten years found Black and Hispanic students to have PhD completion rates of 50% and 58%, respectively (Okahana et al., 2016).

While navigating the preclinical, research, and clinical phases of training, MD-PhD students face unique challenges different from MD-only or PhD-only students (Chakraverty et al., 2018). More MD-PhD than MD students anticipate or experience challenges to balancing training and family life (Kwan et al., 2017). Students also find that the tripartite model of MD-PhD dual-degree programs in the U.S. and Canada creates challenges, having to navigate two transitions between training phases (Bossé et al., 2011; Chakraverty et al., 2018), which most students in MD-only or PhD-only programs do not experience. Among the challenges experienced by MD-PhD students having to transition between the phases are time away from the clinical environment, which could impact students’ preparedness for clinical clerkships (Goldberg & Insel, 2013) as well as a lack of desired mentoring (especially mentoring by MD-PhD faculty), a perceived lack of curricular integration and of awareness of phase-specific cultural differences, and difficulties assimilating with other trainees during the research- and clinical-training phases, who are not from their original cohort of peers (Chakraverty et al., 2018).

Large national cohort studies have examined educational experiences of MD-PhD students as well as variables associated with MD-PhD enrollment (Jeffe et al., 2014b), attrition (Jeffe et al., 2014a), and graduation (Andriole et al., 2008). Individuals who reported participating in high school and college laboratory research apprenticeships, and who highly valued research and finding disease cures as the most important reason to study medicine were more likely to enroll in MD-PhD programs, demonstrating alignment of students’ attitudes and interests with MD-PhD program goals (Jeffe et al., 2014b; Tai et al., 2017). Students who planned substantial career involvement in research at graduation were more likely to be MD-PhD program graduates than all other-MD program graduates; controlling for other variables in the regression model, women and URM students were less likely to graduate from MD-PhD (vs. other-MD) programs (Andriole et al., 2008). In another study of 2,582 MD-PhD program enrollees, 1,885 (73%) had completed the MD-PhD program, 597 (23%) dropped out of the program but completed the MD, and 100 (4%) left medical school entirely (Jeffe et al., 2014a). Although students who enrolled in MD-PhD programs at medical-school matriculation and planned substantial career involvement in research at that time were less likely to leave the MD-PhD program, students who had lower Medical College Admission Test scores, attended medical schools without NIH NIGMS MSTP-funded MD-PhD programs, and were older at matriculation were more likely to leave their MD-PhD program. Notably, women and URM students were neither more nor less likely to leave the MD-PhD program and graduate with only an MD degree (Jeffe et al., 2014a). Students’ MD-PhD program satisfaction was reported to be higher at the beginning of the program and lower during the research phase, due to the unpredictability of time to complete the PhD (Ahn et al., 2007).

Although research has examined challenges faced by potential MD-PhD program applicants (Kersbergen et al., 2020) and by MD-PhD students during their training as described above, to our knowledge, no study has examined the reasons why MD-PhD students leave the program before completing their training using a qualitative research approach. Qualitative research can help explain the decision-making process of individuals (Marshall & Rossman, 2006), adding to our understanding of reasons for leaving the program from participants’ perspectives of their personal experiences. We examined attrition from MD-PhD dual-degree programs using a lens of integration and interaction (Kong et al., 2013) to better understand why some U.S. MD-PhD students ultimately discontinued their training.

## Methods

The data for this paper were collected for a larger qualitative study (Transitions in the Education of Minorities Underrepresented in Research) conducted in the U.S. between 2010 and 2014. This larger study examined training experiences of doctoral students and postdoctoral trainees planning to pursue careers in the biomedical-research workforce to identify factors that served to facilitate or impede progress along this career path (Andriole et al., 2015; Chakraverty, 2013; Chakraverty et al., 2018; Jeffe et al., 2014a; Jeffe et al., 2014b; Tai et al., 2017). In all, we conducted 217 interviews with PhD, MD, and MD-PhD students, postdocs, physician-scientists, and faculty in U.S. higher education biomedical-science PhD programs and in MD-PhD dual-degree programs in U.S. medical schools.

Methodological considerations for conducting a qualitative study were governed by the aims of the larger study to more deeply understand participants’ reasons for considering doctoral-level training in the biomedical sciences in pursuit of a research career and for attrition from MD-PhD training specifically, if applicable, which is the focus of the current study. Using a phenomenological approach, we examined how participants made their decisions to enter or leave their training programs (Marshall & Rossman, 2006). Semi-structured, in-depth interviews allowed us to gather detailed narratives to learn more about all participants’ decision-making processes to enter and either complete or leave their doctoral training (DiCicco-Bloom & Crabtree, 2006). Although this paper focuses on attrition from the MD-PhD program, we also analyzed data for these participants’ reasons for enrolling in the MD-PhD program, to gain a more holistic understanding of their experiences and decision-making processes.

### Data Collection and Analysis

#### Study sample and eligibility

Following Institutional Review Board approval at the University of Virginia and Washington University in St. Louis, we purposefully sampled (Marshall & Rossman, 2006; Miles & Huberman, 1994) U.S. public and private higher education institutions offering biomedical-science PhD degrees and medical schools with dual-degree MD-PhD programs. We sought to interview individuals training for or currently engaged in biomedical research; we also wanted to interview MD-PhD program trainees who dropped out of their program before graduation. We included higher education institutions with the Carnegie classification (The Carnegie Classification of Institutions of Higher Education, n.d.), indicating high or very high research activity. Deans and department chairs disseminated information about the study with our contact information, using emails, announcements, posters, and flyers. We also recruited participants through snowball sampling (Bogdan & Biklen, 2007; Sadler et al., 2010), asking current participants if they would be willing to share our contact information with their colleagues or other students in their program, as well as with individuals who had left their program, and to encourage them to participate in this study. We scheduled phone interviews with individuals who contacted us expressing an interest to participate.

Of 217 participants interviewed in the larger study, 29 students were then currently enrolled in an MD-only program, 20 in a PhD-only program, and 68 in an MD-PhD program; in addition, 25 participants were postdoctoral trainees at the time of the study. Participants no longer in school included 56 faculty, 14 non-scientists, 4 scientists outside academia, and one participant who dropped out of the MD-PhD program before completing either degree. For the current study about MD-PhD program attrition, anyone who had once enrolled in an MD-PhD program but did not complete it was eligible to participate. Overall, seven participants had been enrolled in dual-degree MD-PhD programs but subsequently discontinued MD-PhD training, six of whom continued their training for the MD. The current analysis examines the training experiences of those seven participants and reasons for discontinuing MD-PhD training, which was a specific aim of the larger study.

#### Semi-structured interviews

A semi-structured interview format allowed us some flexibility in asking question better tailored to an individual’s life experiences (Cohen & Crabtree, 2006), although we asked everyone a basic set of questions, (Table 1). Each participant completed one, 45-60 minute semi-structured telephone interview following their informed consent. The interview questions were developed based on the overall study aims, one of which focused on reasons for MD-PhD attrition. The interview protocol and questions were developed by the principal investigators and co-investigators based on their knowledge of gaps in the literature and understanding of the field; interview questions were reviewed by content experts and pilot tested before the initiation of data collection.

Specially trained interviewers, including faculty and PhD students on the research team, conducted interviews for this study. Demographic data such as age, sex, race/ethnicity, and current program were collected at the beginning of each interview. Interviews were audio-recorded with permission, transcribed verbatim through a professional company, and assigned an alpha-numeric code prior to analysis. For this aim of the study focusing on MD-PhD students’ reasons for leaving the MD-PhD program, in-depth interviews were conducted to gain insight into participants’ backgrounds, experiences, reasons for enrolling in MD-PhD programs, and when and why they discontinued their MD-PhD training through their own narratives (Kvale & Brinkmann, 2009). We sought to identify aspects of the training that might have been particularly problematic for these participants. Probing questions were asked based on participants’ responses. All the authors have directly conducted or aided in medical education research for varying lengths of time. At the end of each interview, participants were asked to broadly share information about the study in their professional and personal networks, so that people from a wider network would become aware of this study using snowball sampling (Bogdan & Biklen, 2007; Sadler et al., 2010).

#### Analytic strategy

Each interview transcript was open-coded by two authors, both for narratives about their reasons for enrolling in the MD-PhD program and for leaving the program. The coders created a single codebook after discussing and resolving disagreements about codes, compiling all the codes into a final list that was used to reanalyze all the interviews. Since attrition MD-PhD program attrition is a relatively understudied topic, codes were based on participant transcripts rather than existing literature. Using an inductive, thematic approach as the primary analysis strategy (Miles & Huberman, 1994; Pope et al., 2000) and the constant comparative method of coding (Glaser & Strauss, 1967), the codes were systematically organized into themes (Thomas, 2006). Themes that emerged from the analysis are presented if experiences fitting in a theme were discussed by multiple participants. Although some reasons described during the interview were unique for a participant, we elaborate only on those recurrent themes and experiences that were common across multiple participants. Both coders were mindful of the fact that their worldviews and positionalities could differ from those of the participants, interviewers, and from each other, which could influence how the interviews were conducted and data were analyzed (Antin et al., 2015). Both coders were a part of the interview team and are educational researchers with a background in higher education and medical education research; they used a reflective journal, recording memos to document their coding decisions during analysis and acknowledge any disconfirming evidence. The coders also consulted with each other to ensure agreement on coding. They resolved coding disagreements through a discussion and consensus. The coding and analysis process lasted roughly seven months. We present representative quotes that exemplified the emergent themes, adding content in brackets to clarify a participant’s narrative. We used pseudonyms for those participants whose results are described in this manuscript.

## Results

Of the seven participants who had left the MD-PhD program before completion, two had completed their MD training and held academic-medicine faculty positions at the time of their interview; four were still in medical school completing the MD degree, and one was completing a PhD in Education (Table 2). Since the sample size was small, our findings are exploratory; we did not expect to reach data saturation, a stage when no new themes emerge as a result of further data collection (Faulkner & Trotter, 2017; Glaser & Strauss, 1967). Although there were similarities in the reasons that participants gave for entering MD-PhD training, each participant described slightly different circumstances and stages during which they left MD-PhD training.

### Why participants entered MD-PhD training?

We asked participants what inspired them to pursue MD-PhD training in the first place. All seven participants provided reasons that included both a desire to help people on a day-to-day basis through clinical practice and to more deeply engage in research. Having the MD-PhD dual degree was perceived as a way to broaden research opportunities to participate in clinical and other types of research as well as get access to patient populations. For all participants, the desire to pursue a research career grew from undergraduate research opportunities that they had experienced; such opportunities led to publishing and presenting at conferences, networking with established researchers, and getting to know “what their careers were like” (Debbie). Ben had “a pretty thorough research experience” in college where he “worked every summer in the research lab” and had already published research by the time he finished college. Aaron described an undergraduate mentor who was “a very good chemist and a wonderful teacher” who taught him “how research is done and the rewards of doing research.” A fulfilling college research experience also provided participants with the skills to handle research responsibilities, independently decide what experiments to conduct, and develop ownership of the work—factors that made participants consider studying for an MD-PhD.

In college, it became much more concrete, this idea that I wanted to do research and medicine, and try and incorporate the two. The experience gave me this little niche to be working in and got me really excited about what scientists do. (Eva)

During college, participants reported having opportunities to give presentations at national conferences and to gain insight into clinical experiences by shadowing physicians, and volunteering to help children with special needs. Such experiences shaped one’s desire to pursue MD-PhD as opposed to MD-only or PhD-only. Eva, who wanted to combine medical training with research training shared, “as much as I like the research and thinking about science, I wasn’t cut out to just be in the lab all the time by myself.”

Participants were also influenced by undergraduate mentors who provided hands-on research experience by “letting me have my own little section of the project.… He said, ‘Here's a part of the project. I want you to figure this thing out.’ I think that’s what really sparked my enthusiasm for basic science” (Francesca). Overall, Eva realized that receiving both the MD and PhD would help “produce new knowledge and provide independence” and the “thrill of discovery.” College mentors also helped select and apply to MD-PhD programs and provided information about how one could combine patient care and research if they had the dual MD-PhD. Gerald noted, “The premed adviser at the house [dormitory] was an MD-PhD. He did have a relatively big influence on my decision to pursue MD-PhD.” A dual-degree meant that “I don’t have to give up one side of something that I find exciting and want to explore.”

Participants were motivated by a combination of positive research experiences and personal reasons to pursue the MD-PhD. For example, Debbie shared,

After college I worked as a research technician in a lab studying HIV, and I worked with a lot of physicians who also did research. I sort of liked the idea of the variety in their careers, so I was looking into programs that would allow me to see patients plus do research, and that was how I decided to apply to [the] MD-PhD program.

Personal or family reasons also was a motivation for pursuing MD-PhD. Gerald reasoned, “my grandma was often sick in the nursing home. Going back and forth from the hospital to the nursing home to home. I wanted to help people like her.”

In summary, participants wanted to pursue MD-PhD to be able to work in two worlds—clinical practice and research. Clearly, very positive college research experiences, mentorship, and personal reasons also played big roles in participants’ decisions to pursue the dual MD-PhD degree. And for some, the icing on the cake was the lure of opportunities to participate in a variety of professional activities that they could enjoy as an MD-PhD. So what happened to make these individuals change their minds?

### Why participants left the MD-PhD training?

Aaron and Eva left their MD-PhD program at the end of second year without starting the PhD training phase at all; the other five participants completed some of their PhD training before discontinuing the MD-PhD program (Table 2). Once in the program, the influence of positive role models and opportunities that drew candidates to the program was weakened by a variety of factors. Four recurrent themes emerged from the data with regard to participants’ reasons for leaving the MD-PhD program without completing the requirements for both degrees (Table 3), which we describe below.

#### Declining interest in research

Three participants (Aaron, Debbie, and Eva) shared that although they joined an MD-PhD program to pursue research as well as clinical care, their interest in research and earning a PhD declined shortly after starting the program, which contributed to their decisions to leave the program. At the time of the interview, Aaron was a faculty of clinical research at a medical school and in his sixties (describing experiences from his twenties), and Eva was a second-year medical student in her twenties. Yet, both shared similar experiences of a decline in interest in research following the first few research rotations during their MD-PhD training. Both left their MD-PhD program at the end of their second year of medical school, without formally starting PhD training at all, although both had pursued summer research opportunities during medical school.

For both, it was a combination of being exposed to interesting clinical problems during MD pre-clinical phase, summer rotations shortly after that did not yield research, and a declining interest in research, where “All of a sudden, the PhD just didn’t seem like the thing that I wanted to do anymore, even though when I applied a year and a half ago, I was super excited about it” (Eva). In both cases, lab rotations did not fit research interests, creating doubts about how attractive the PhD would be. Both had an enriching research experience in college that contributed to their decision of doing an MD-PhD. However, once the program started, the excitement:

sort of fizzled. I couldn’t really find something that would keep me interested in that same way. … I was less than thrilled about what I was doing. That was why I first started questioning what I am looking to get out of this. (Eva)

Aaron did not want to put his clinical training on hold after two years and “take off three or four years to go into a lab when I didn’t have a hot project that I was totally enthused about, having had my project from the prior summer sizzle out.” He felt frustrated “not having something [in research] that I had a lot of enthusiasm for. I’d heard about all these fascinating clinical issues and conditions and examined just a couple of patients and thought that was very exciting.” That led him to gravitate towards only the MD degree. The structure of MD-PhD program felt illogical, “giving you the preparation for going into clinics and then saying, ‘Okay, we’ll put that on hold for four years and let’s go do research,’” Aaron shared. At the end of two years, when their MD-PhD cohort split with the rest of the MD classmates, he decided to only continue his clinical training.

This was also largely as a result of positive pre-clinical experiences where both Aaron and Eva learnt a lot from the preceptorship in the first year, an elective mentored experience where one was paired up with a physician to shadow and be involved in doing interviews and physical exams with patients. Debbie, who left after the third year of the MD-PhD program (after two years of medical school and one year of PhD) did that due to the uncertainty of producing research results and lengthy training for the PhD. At the start of her MD-PhD program, she “loved the medical school curriculum and working with other medical students.” However, when she started her PhD training at the beginning of third year, she did not like research as much and felt underprepared for research compared to her MD-PhD peers. She was “leaning more towards medicine” and “didn’t quite fit the MD-PhD profile.” She shared being “not excited everyday by going to the lab, the way I am excited to go to the hospital every day. I just felt like I was missing something. I was unhappy and frustrated doing research.” She realized that she enjoyed clinical training more than research, did not feel as prepared or enthused about getting a PhD by the third year, and felt out of place in the research lab. Like Eva, Debbie would prefer conducting research during residency rather than continuing training for the PhD and ultimately being responsible for running a research lab as a principal investigator.

#### Isolation and lack of social integration during the different training phases

Social integration broadly describes the ways in which MD-PhD students were able to assimilate into the different cultures during the various training phases. Students described the challenges they experienced and ability to interact with other MD-PhD students as well as with PhD-only and MD-only students during the respective research- and clinical-training phases. Five participants (Ben, Carrie, Eva, Francesca, and Gerald) described challenges in integrating socially in different phases of the MD-PhD program that eventually contributed to their decision of leaving the MD-PhD program. Lack of both family and peer interaction contributed to feelings of isolation.

##### Family interaction:

There were feelings of isolation due to living far away from family and a cohesive community with which they were familiar. Eva shared that eventually, the novelty of MD-PhD went away and stress related to how long the training was going to take set in. None of the seven participants had an immediate family member in medicine, and four of them were first-generation college students. Having a physician parent might have provided participants with more opportunities and resources to understand and feel comfortable with the demands MD-PhD training. There was a “disconnect in how much my family understands about what I’m doing here at school,” shared Eva. Families sometimes did not understand the academic pressures or the purpose of undergoing such a long training. Although participants reported they did not get much family support while pursuing MD-PhD, Ben shared that he received family support when he decided to leave the program.

##### Peer interaction:

Isolation due to poor peer interactions started as early as by the second year of MD-PhD training. Socialization opportunities during PhD were inadequate and not as fulfilling, making “the cultural transition from medicine to science a very hard one” (Francesca). It was difficult to mingle with PhD-only students who had already gone through a year of classes and lab rotations with other PhD students and had formed their groups. Participants felt like outsiders in the PhD program. Francesca felt frustrated interacting “with the same five people all day, every day. I was feeling isolated from other people.”

I loved interacting with the patients. I loved the immediacy of medicine. It was a slow realization for me over the last year and a half that I was in the lab that I was much more passionate about the day-to-day work of medicine than I was about the day-to-day work of science. I think part of it as the sort of solitary nature of it [lab research]. I feel like I’m more of a people person than I could be while I was in the lab. (Francesca)

Lack of a social circle was also a challenge, Gerald shared not having “close friends who were doing it [completing MD-PhD training]. Maybe that would have given me more insight into the day-to-day life and might have swayed me a different way.” When a large cohort of MD-PhD students split up to go to different departments during their PhD training, daily interactions with fellow MD-PhD students decreased for him.

Ben felt like being in a difficult environment and a “strange, no-man’s land” to work where neither the MD nor the PhD students considered MD-PhD students one of their own.

[It was like a] cold war between the MD and the PhD faculty at a medical school. The PhDs feel that their degree is of slightly higher rank than an MD and should be treated thus. In a medical school, the MDs insist that [they rank higher]. Both sides feel that they should be in charge, and the other ones are the secondary people. (Ben)

Carrie felt that it would be less stressful if she left PhD training since she had not met a single MD-PhD graduate who was happy. MD students “looked down” on MD-PhD students, considering them to be poor clinicians “because you split your time doing research” and “the PhD did not help in the clinic,” she shared, adding that fellow PhD students did not consider MD-PhDs as serious researchers, saying that MD-PhD students’ “research training was watered down.” Both Carrie and Francesca felt that students in each phase were territorial. Carrie described an “us-against-them mentality”—where MD students considered the PhD-phase of the MD-PhD program as “getting a vacation,” and PhD students were of the opinion that “this isn’t med school where people will hold your hand and spoon-feed you what you need to know.” Francesca felt the cultural transition to the PhD program and the several-year-long gap in medical training were formidable challenges.

In addition to unsatisfactory peer-interactions, Eva eventually realized she enjoyed the daily interactions she experienced working in a hospital more than while conducting research, “which is very much sort of intellectual and introverted. What changed most were the internal factors about what I want out of my career and my life.”

#### Suboptimal PhD-advising experiences

Three participants (Ben, Carrie, and Gerald) described several challenges related to inadequate mentoring and PhD-advising that contributed to their decision to leave the program. Lack of adequate mentoring during a very regimented MD-PhD training was a widely discussed challenge. Those who left the program described the mentoring they received as minimal, inadequate, sparse, and hands-off. Advisers did not always help in coping with the stress of a long training process, especially during PhD when students had already spent a few years in the program. This was especially discouraging for first-generation students who had received no guidance at home. “Nobody asked if there were problems down there [in the PhD lab] until I did my resignation letter, and then they’re like, ‘Oh, well, what can we do to get you to stay?’ At this point, nothing,” shared Carrie.

Students lacked the bigger picture of what an MD-PhD would be doing ten years down the line, the MD-PhD’s perspective on career development and how to handle training challenges, which could only be provided by MD-PhD advisers (compared to advisers with an MD-only or PhD-only degree). Female MD-PhD students sought female MD-PhD advisers to understand how to achieve work-life balance, who were even rarer to find. Overall, MD-PhD advisers were hard to find.

In addition to bad experiences with PhD advisers and lack of MD-PhD advisers overall, a positive experience with an MD preceptor actually steered students away from a PhD towards an MD-only program. Overall, conflicts arose when adviser and student’s professional goals and values did not match. This happened when a PhD adviser only trained students to become the next generation of principal investigators in a basic science research lab, while that was not the goal for an MD-PhD student. This mismatch made the relationship uncomfortable, especially when advisers “expressed negative opinions of medical students” and treated them more like an employee, shared Gerald.

Ben shared that many PhD advisers were “hostile to the fact that I was an MD-PhD student” and “wore a chip on their shoulder all the time over their position vis-a-vis the doctors. That was just generally a difficult environment to function in.” PhD advisers especially made a difference in a good or bad way because the PhD training process itself was long, with years of research not always yielding publishable results. Given this uncertainty, having young, inexperienced, and pre-tenure PhD advisers further posed challenges, created negative experiences, and discouraged MD-PhD students from completing a PhD. Ben eventually lost his PhD support and was “kicked out against my will for having made inadequate progress” in research. He shared that PhD advisers had “full authority to judge on any criteria they want whether someone has made adequate progress,” and there was no legal defense against that, even if certain committee members did not agree with the decision to expel a student. Often, when a PhD collaboration between faculty and MD-PhD student did not work out, it was difficult to identify another PhD adviser because of smaller MD-PhD programs (compared to PhD-only programs) with fewer available faculty.

When there was lack of MD-PhD advisers, having a better adviser in one phase could disproportionately shift the balance and make students want to complete that part of the training. Gerald had issues working with his PhD adviser, but his MD mentor was very supportive and “willing to meet with me any time to discuss how things are going in medical school, getting back into study habits for medical school after being out for four years.”

#### Unforeseen obstacles to completing PhD research requirements

Four participants (Ben, Carrie, Francesca, and Gerald) described various unforeseen circumstances that they experienced while completing research requirements during the PhD-phase, which contributed to their decision to leave MD-PhD training. Gerald was in his sixth year (two years of MD and four years of PhD) when he left MD-PhD training. At the beginning of PhD training, none of the lab rotations culminated into a fruitful experience to facilitate completing PhD. Sometimes, “animal models did not work,” forcing one to abandon experiments after many years of effort.

It [the animal model] was still expressing the gene. It was still making the protein, but the phenotypes that…. were no longer there. After several generations of outbreeding were still not there. I was the only person using this model. (Gerald)

Experimental failures created tension between Gerald and committee members because “there was kind of a disconnect between what the rest of my committee expected and what my mentor was able to support.” Even when the program advised to start a new project, it was not possible; Gerald’s PhD adviser “didn’t really have the time or the energy to get that [a new project] off the ground.” Lack of time became a challenge.

I had two weeks to write a completely detailed proposal on this new project. Based on my experience just working with the phenotype and the amount of time and energy that went into that, then looking at [how to] be able to get this new project finished, it would’ve required even more time and energy. It no longer seemed feasible to me. (Gerald)

The possibility of joining a different lab was also eliminated due to time constraints. Francesca, who was in her eighth year of training (two years of MD and six years of PhD) when she left the program, continued to lose more time when the PhD adviser moved to a different university and there were facility-based technical problems.

They constructed [for] us a containment facility instead of a clean room for some of the work, so the airflow was backwards, and all the cultures got contaminated for months. I think I probably lost about nine months with the move and getting all these things straightened out again. (Francesca)

As a result, she felt that the PhD training was tedious and “the things I love about research are sort of hard to vet.” She added, “after six years of doing something, if you’re still spending a lot of time optimizing it, you’re not necessarily learning anything from that. It’s just sort of rote, repetitive work.” Ultimately, the length of time needed to complete MD-PhD training created other personal challenges that contributed to participants’ decisions to discontinue the MD-PhD program. For Ben and Carrie, the lengthy training time, especially during PhD, deterred them from raising a family. Carrie added, “The system penalizes individuals who need to take a break in between their training.”

## Discussion

This study reported results of an analysis of interview data that were collected for a larger qualitative study of training experiences and career decisions made by individuals pursuing biomedical-research careers. Although findings reported here reflect perspectives of only seven individuals who left the dual MD-PhD program before completion, their narratives provide a deeper understanding of reasons for discontinuing training—reasons that have not been captured in surveys (Ahn et al., 2007) or even in large, retrospective, national-cohort studies (Jeffe et al., 2014a).

Six of the seven former MD-PhD students finished medical school and completed requirements for the MD degree. Although five left the MD-PhD program within 2-3 years of matriculation, two left after six or more years of training due to extenuating circumstances related to their PhD advisers. Participants’ narratives included details of their reasons for leaving the MD-PhD program. Overall, four recurrent themes emerged from the data, including: 1) declining interest in research, 2) isolation and lack of social integration during the different training phases, 3) suboptimal PhD-advising experiences, and 4) unforeseen obstacles to completing PhD research requirements. Interestingly, analysis of data from 48 then-current MD-PhD students who participated in the larger study also reflected two of the same challenges, specifically, isolation and the lack of social integration during different training phases due to the need to transition between phases, and suboptimal PhD advising; the other challenges experienced by then-current MD-PhD students included a perceived lack of curricular integration as well as cultural differences between the MD and PhD phases of training (Chakraverty et al., 2018). The current study expands upon findings from that earlier study to examine factors that compelled some students to leave their MD-PhD training altogether, and during which phase they left the program.

Despite the small sample size, study findings add to our understanding of the challenges of completing the requirements for the dual MD-PhD as part of a lengthy and disjointed training program. Participants described a complex interplay between students, faculty, and the administrators, resulting in experiencing difficulties with assimilation and immersion into different MD-PhD program cultures through which they transitioned during training. Prior to entering the MD-PhD program, each of these participants reported having had substantial and positive research experiences in college. However, although potentially crucial for decisions to enroll in MD-PhD programs (Jeffe et al., 2014b), and even to apply for and be accepted to medical school in general (Andriole et al., 2015), having substantial, positive college research experiences was not enough to keep these participants in the MD-PhD program. Most of those who left PhD training were still interested in pursuing research in the future, but they did not feel the need for a PhD. According to the 2014 NIH Physician-Scientist Workforce Working Group Report (Feldman, 2014), some of the contributors to a leaky workforce include an aging physician-scientist population, long and poorly compensated training, and fewer role models (especially for women and URMs).

For participants who left their MD-PhD training program, the MD-PhD dual degree ultimately did not seem to enhance their career prospects as a researcher; an MD degree alone was deemed sufficient to conduct clinical research. From these participants’ perspectives, what mattered most for research were the grants that people were awarded, the publications, and even faculty appointments. From their point of view, the PhD degree did little to enhance what an MD could offer.

The literature, however, shows benefits of MD-PhD program participation for sustaining and promoting medical students’ intentions to pursue full-time academic-medicine careers (Jeffe et al., 2008) and acquiring full-time faculty appointments (Andriole & Jeffe, 2016). More than half of MD-PhD graduates in a national cohort of medical school matriculants received academic-medicine faculty appointments (Jeffe et al., 2012) compared with only 18% of MD graduates (Andriole & Jeffe, 2012; Jeffe et al., 2012). In addition, compared with MD graduates in this same cohort, MD-PhD graduates were more likely to receive each of individual postdoctoral research fellowships (F32) awards, career development (mentored-K) awards, and research project grants (R01) in models controlling for a number of demographic, research related, and academic variables (Jeffe & Andriole, 2018). Moreover, MD-PhD program graduation also has been shown to be a significant mediator of observed racial/ethnic disparities in mentored-K awards in this national cohort (Andriole et al., 2017).

The most prominent finding of the current study is that most participants who left MD-PhD training did so during the PhD-phase. Prior research on PhD-program attrition suggested that PhD training, including in MD-PhD dual-degree programs, was particularly problematic for students who could not integrate well with their peers during this PhD phase of the program (Golde, 2000). Some of the factors related to PhD-program attrition include social isolation (Ali et al., 2007) and the nature of advising, including perceptions of autonomy and relatedness during dissertation (Burns & Gillespie, 2018). Doctoral faculty tend to attribute causes of doctoral-student attrition to student-level factors, often not acknowledging the role of departmental factors (Gilmore et al., 2016). This is despite evidence that the departmental climate and advisers play an important role in their students’ abilities to complete or not complete their training (Devos et al., 2016). Although other factors such as experiencing mental health and adjustment issues due to the impostor phenomenon (where doctoral students feel like intellectual frauds) have not been documented in the literature on doctoral students’ attrition, such factors have influenced student experiences during doctoral training (Chakraverty, 2019; 2020a, 2020b, 2020c).

Findings from student participants who were still completing requirements for the MD-PhD program identified the importance of more advanced students serving as peer mentors (Chakraverty et al., 2018). Both then-current MD-PhD students in that study and MD-PhD program drop-outs mentioned the critical need for good faculty mentors, and especially MD-PhD mentors who overcame the challenges they faced as students completing MD-PhD training. Both faculty and peer mentors who have faced similar challenges can provide unique insight into what this long and complex training entails (Chakraverty et al., 2018). Notably, none of the participants who dropped out of the MD-PhD program mentioned having supportive peer mentors.

Purposefully building mentoring relationships might help MD-PhD students stay the course during challenging times. Such mentorship groups could involve an MD-PhD student, more advanced MD-PhD students or recent MD-PhD graduates, and faculty, because transitioning between MD-PhD program phases is particularly challenging for these students (Chakraverty et al., 2018).

MD-PhD students who left the program described many challenges assimilating into each program phase due to the disjointed structure of MD-PhD training that did not allow specific program support for socialization and integration. Students transitioning from MD to PhD phases were expected to already know the values and culture of PhD training as well as what was expected of them during PhD training to be able to blend in, something that participants did not always know. Such seamless integration between the different phases was challenging for the MD-PhD students interviewed, but also may require specific integration strategies through re-immersion programs (Goldberg & Insel, 2013) and career-development programs (Ciampa et al., 2011) at each transition. We strongly recommend such academic and socialization strategies to facilitate cultural integration within a program that is as complex as the MD-PhD dual-degree program.

Previous research reported that women and URM students were less likely to be MD-PhD program graduates compared with all other MD program graduates (Andriole et al., 2008); however, in a national cohort study of MD-PhD program matriculants, neither gender nor race/ethnicity were independently associated with overall attrition from MD-PhD training (Jeffe et al, 2014a). Our findings show that while evaluating the possible benefits of pursuing the MD-PhD, participants in the present study mostly discussed the disadvantages of a long training time. MD-PhD completion time increased from an average of 6.6 years in 1980 to 8.0 years between 1998 and 2007 (Brass et al., 2010). Such a long training period may itself be a deterrent to program completion, delaying the time to achieve research independence and leading some students to choose clinical practice over research (Gordon, 2012). Notably, however, the time to first R01, the hallmark of research independence, was nearly 2 years shorter from time of graduation for MD-PhD than MD graduates (Jeffe & Andriole, 2018).

### Limitations

This was an exploratory study of a very small sample of mostly White individuals who did not complete the MD-PhD program in the U.S. Given the small sample size and homogeneous demography, the findings are not generalizable to the larger MD-PhD student population, in the U.S. or elsewhere. Although age at the time of starting MD-PhD training was not asked, it is evident that most, if not all started their MD-PhD training in their twenties. Further, two participants in their forties and sixties, both medical school faculty when they were interviewed, recalled their experiences in the MD-PhD program from more than a decade before being interviewed, which could be affected by recall bias; however, their experiences were similar to the other participants who only recently left their MD-PhD program when they were interviewed. Nevertheless, findings provide important insight into the reasons for discontinuing their MD-PhD training through a qualitative examination of MD-PhD student narratives, which, to our knowledge, has never before been undertaken. However, the phenomenon of MD-PhD program attrition needs to be examined in greater detail, with a larger and more diverse sample of MD-PhD students who left the program. In addition, we did not elaborate on thematic reasons that were not reported by multiple people, which does not mean that reasons reported by only one person were not important. Nor does it mean that reasons reported only by one person here would not be reported as a recurrent theme had we had interviewed a larger sample of participants who had left the MD-PhD program. Indeed, only one participant, who was Hispanic, described in detail how her decision to leave the program was influenced by the need to stay close to her family and Hispanic community. Although the racial/ethnic diversity of MD-PhD program graduates increased from 5.0% of graduates from URM groups in 1995 to 9.6% in 2015 (AAMC, 2016), URM representation among MD-PhD graduates is considerably lower than their overall representation of more than 30% in the U.S. population (Colby & Ortman, 2017). Additional research is needed to examine URM MD-PhD students’ reasons for MD-PhD program attrition.

### Implications and future directions

The findings of this study provide a perspective to understand doctoral research capacity building. While capacity building at the micro-level examines how students transition between the various phases of their training and transform into scholars (Lovitts, 2005), capacity building at the macro-level examines the larger-level trends such as increasing demographic diversity and skill building (Trostle, 1992). Overall, building one’s capacity to be an independent investigator should ideally entail structured mentoring and supervision in the relevant content area, developing specialized, transferable skills, as well as professional development and mentoring to learn about a variety of career prospects outside academia. MD-PhD program attrition can have both micro- and macro-level implications. Micro-level implications include costs to funding agencies and MD-PhD programs (Jeffe & Andriole, 2011; Jeffe et al., 2014a), as well as to faculty mentors and students themselves (i.e., in time lost and financial burden). It also has macro-level implications in terms of a reduction in the cadre of highly trained, clinical and translational science researchers. Although based on a small sample size, the fact that most attrition happened at the PhD-training level calls for a deeper examination of the challenges students described herein regarding their experiences during the PhD-training phase of MD-PhD training. Findings shed light on situations and experiences that dissuaded these students from completing their PhD training. We urge future research to more deeply examine how interactions among students, faculty and administrators in various settings, such as classrooms, research labs, and clinics, and between different schools and departments, can help MD-PhD students fully integrate into each new program phase they are entering and to continue in the program to completion.

As women and some racial/ethnic groups are underrepresented among MD-PhD program trainees (Jeffe et al., 2014b), increasing the diversity of trainees in MD-PhD programs might ultimately serve to increase both the size and diversity of the larger physician-scientist workforce to better meet the needs of an increasingly diverse population (Milewicz et al., 2015; NIH, 2014). Examining MD-PhD training experiences through the lens of gender and race/ethnicity should be undertaken in future research with a larger and more diverse sample.

Although greater planned career involvement in research at matriculation was observed to be a predictor of MD-PhD program completion (Jeffe et al., 2014a), we found that extenuating circumstances during students’ training in these programs, and apparently, especially during the PhD phase of training, served to derail some of these students’ aspirations to graduate with the MD-PhD dual degree. Attendance at institutions with MSTP funding has been shown to be beneficial and predictive in terms of MD-PhD program completion (Jeffe et al., 2014a), and students who attended schools supported by MSTP funds especially benefited during their PhD training (Goldstein & Brown, 1997; Jeffe & Andriole, 2011; NIH-NIGMS, 1998). However, students whose research is funded solely by their advisers’/mentors’ grants are at greater risk of dropping out of the program for lack of funding, if the advisers’/mentors’ labs closed because they could not renew their grants in the middle of the MD-PhD student’s training in their lab. Institutional MSTP funding has been found to be predictive of students’ retention in the program (Jeffe & Andriole, 2011) and of faculty appointment among MD-PhD graduates (Andriole & Jeffe, 2016).

## Conclusion

This paper examined interview responses from seven participants in a larger study who left their MD-PhD programs before completing training; two participants had completed the MD and were academic-medicine faculty, four were completing medical school, and one dropped out of medicine to complete a PhD in Education. Participants reported enrolling in MD-PhD programs to work in both clinical practice and research. Very positive college research experiences, mentorship, and personal reasons played big roles in participants’ decisions to pursue the dual MD-PhD degree. However, once in the program, the influence of earlier positive role models and opportunities that drew candidates to the program was found lacking in the MD-PhD program and weakened their resolve to continue to completion. Four themes emerged as reasons for leaving the MD-PhD program: declining interest in research, isolation and lack of social integration during the different training phases, unsatisfactory PhD-advising/mentoring, and unforeseen obstacles to completing PhD research requirements. We conclude that providing better institutional and social support for the timely completion of research and targeted research mentorship are essential to retaining and promoting the success of students during the PhD phase of their MD-PhD program training. The themes that emerged from participants’ narratives in the current study suggest that targeting interventions to improve students’ educational and research experiences, mentorship, and integration into the different cultures of each program phase are crucial for retention of MD-PhD students through to completion of the program. These same challenges arising from having to transition into different phases of the MD-PhD program were described as well in a larger sample of 68 students who were still in training for the dual MD-PhD degree (Chakraverty et al., 2018). Through a deeper examination of reasons for attrition, MD-PhD programs can find ways to improve training experiences and improve student retention; this can strengthen the biomedical-research-workforce capacity.

## Figures and Tables

**Table 1.** Interview Questions Asked of Participants

INTERVIEW QUESTIONS
1. Tell me about your current program. What phase of your program are you in?
2. How did you decide to pursue an MD-PhD degree? What were the factors you considered when applying to programs? What was the MD-PhD program structure and how did moving between phases of the MD-PhD program work?
3. Tell me about your expectations coming into the program. In what ways has the program met, or differed from, your expectations?
4. Beyond programmatic rigor, tell me about any specific barriers that you felt you needed to overcome to succeed in the MD-PhD program.
5. Could you share the reasons you decided to discontinue MD-PhD training?
6. Tell me about your interactions with other people in the MD-PhD program.
7. Tell me about your experiences with advising or mentoring, from professors or peers in your MD-PhD program.
8. Who is your support system outside of your program?
9. Looking back at your own past experiences, were there one or two things that, had they happened differently, might have led you to choose some other educational path?

**Table 2.** Participant Demographics, Timing of MD-PhD-program Attrition, and Status at Time of Interview

PSEUDONYM	GENDER, RACE AND AGE (YEARS)	TIMING OF MD-PHD- PROGRAM ATTRITION	POSITION AT THE TIME OF INTER- VIEW
Aaron	Male White 62	After 2^nd^ year to complete MD degree only. Only did research rotations for PhD.	Medical school faculty
Eva	Female Hispanic 24	After 2^nd^ year to pursue MD degree only. Completed 2 research rotations in previous summer as a part of the MD-PhD program.	2^nd^ year MD student
Ben	Male White 30	After 3^rd^ year (the MD and PhD training phases were mixed) to pursue PhD in Education. Thus, he completed neither the MD- nor the PhD-degree requirements of the MD-PhD program	PhD student in education
Carrie	Female White 45	After 3^rd^ year (including 2 years of MD and 1 year of PhD) to complete MD degree only. Also pursued and subsequently completed a PhD separately in education.	Medical school faculty
Debbie	Female White 28	After 3^rd^ year (including 2 years of MD and 1 year of PhD) to pursue MD degree only.	4^th^ year MD student
Gerald	Male White 30	After 6^th^ year (including 2 years of MD and 4 years of PhD) to pursue MD degree only.	4^th^ year MD student
Francesca	Female White 31	After 8^th^ year MD-PhD (including 2 years of MD and 6 years of PhD) to pursue MD degree only.	3^rd^ year MD student

**Table 3.** “Why participants left MD-PhD training?”: Frequency for Each Theme

PSEUDONYM	DECLINING INTEREST IN RESEARCH	ISOLATION AND LACK OF SOCIAL IN- TEGRATION DURING THE DIFFERENT TRAINING PHASES	SUBOPTIMAL PHD- ADVISING EXPERIENCES	UNFORESEEN OBSTACLES TO COMPLETING PHD RESEARCH REQUIREMENTS
Aaron	X			
Eva	X	X		
Ben		X	X	X
Carrie		X	X	X
Debbie	X			
Gerald		X	X	X
Francesca		X		X
**Total frequency**	**3**	**5**	**3**	**4**
